# Tuberculosis and Type 2 Diabetes Mellitus: An Inflammatory Danger Signal in the Time of Coronavirus Disease 2019

**DOI:** 10.1093/cid/ciaa747

**Published:** 2020-06-13

**Authors:** Robert J Wilkinson

**Affiliations:** 1 Francis Crick Institute, London, United Kingdom; 2 Department of Infectious Diseases, Imperial College, London, United Kingdom; 3 Wellcome Center for Infectious Diseases Research in Africa, Institute of Infectious Disease and Molecular Medicine and Department of Medicine, University of Cape Town, Cape Town, Republic of South Africa

**Keywords:** tuberculosis, diabetes mellitus, COVID-19, inflammation, risk factors

**(See the Major Article by Eckold et al on pages 69–78.)**

There is an association between type 2 diabetes mellitus (DM) and tuberculosis (TB) [1]. The association is becoming more significant due to the epidemiological transition as DM is growing rapidly in settings where TB is common. DM increases the risk of developing TB and is also associated with severe cavitating disease and adverse treatment outcomes, including death [2–5]. TB increases insulin resistance and stress-induced hyperglycemia that may revert to normal during treatment [6, 7]. Testing for DM in TB patients is thus recommended, with confirmatory tests after 2–3 months of TB treatment initiation [8].

The risk of diabetics contracting infections has long been attributed to the hyperglycemic environment that favors immune dysfunction (eg, impaired neutrophil function, depression of the antioxidant system, and humoral immunity), micro- and macroangiopathies, neuropathy, a decrease in the antibacterial activity of urine, gastrointestinal and urinary dysmotility, and a greater number of medical interventions [9]. However, it is increasingly appreciated that DM itself is an inflammatory disorder that may therefore interact with infection in more complex ways [10].

Transcriptomic profiling of whole blood is a technique that has been widely applied and proven insightful in many infectious and inflammatory disorders [11]. Active TB has a transcriptomic signature dominated by a neutrophil-driven type 1 and 2 interferon-inducible gene profile. The transcriptomic signature relates to disease extent and resolves during successful treatment [12]. The differentiation of active from latent TB and other conditions may be aided by transcriptomic profiling [13]. The exaggerated inflammation that characterizes human immunodeficiency virus (HIV)-TB–associated immune reconstitution inflammatory syndrome is triggered by Toll-like receptor and inflammasome signaling [14]. Transcriptomic signatures may predict progression of, or detect, subclinical TB [15–17].

In this issue of the *Clinical Infectious Diseases*, Eckold and colleagues present an analysis of the effect of DM and intermediate hyperglycemia on the TB whole blood transcriptomic signature [INSERT REF.]. Samples were collected from active TB patients with DM ( hemoglobin A1c [HbA1c], ≥6.5%) or intermediate hyperglycemia (IH; HbA1c, 5.7%–6.5%), TB-only patients, and healthy controls in 4 countries: South Africa, Romania, Indonesia, and Peru. Differential blood gene expression in 249 participants was determined by RNA sequencing. DM increased the magnitude of gene expression change in the host transcriptome in TB, notably showing an increase in genes associated with the innate inflammatory response and a decrease in the adaptive immune response. Patients with intermediate hyperglycemia and TB exhibited blood transcriptomes that were more similar to those of DM-TB patients than of patients with only TB. Both DM-TB and intermediate hyperglycemia-TB patients had a decreased type I interferon response and a relatively stronger neutrophil component relative to TB-only patients, suggesting skewing of the TB transcriptome phenotype. Such immunological dysfunction is also present in individuals with intermediate hyperglycemia, showing that altered immunity to TB may also be present in this group. The authors conclude that TB disease outcomes in individuals with IH diagnosed with TB should be investigated further.

The study has the advantage of power and of validation across populations. Limitations are that HbA1c is a relatively insensitive test for DM. It is known that TB itself associates with transiently impaired glucose tolerance, which may even be in the frank diabetic range, yet which may resolve during TB treatment [7]. However, the proportion of cases of IH that fell into this category was not investigated longitudinally. Sex influences type 1 interferon and neutrophil transcript abundance, and there were some very substantial gender differences between groups, ranging from 18% to 90% males.

This publication coincides with the world grappling with the coronavirus disease 2019 (COVID-19) pandemic. Are there inferences that can be made from this very detailed study of susceptibility to TB conferred by diabetes? Male sex and diabetes have been identified in virtually every study as risk factors for severe COVID-19 infection, associated in the largest study to date with an adjusted hazard ratio for in-hospital death of 1.99 (male sex), 1.50 for controlled DM (HbA1c <58 mmol/mol), and 2.36 for uncontrolled DM [18]. The pulmonary innate immune response in COVID-19 patients with severe disease implicates a macrophage subpopulation with an interferon-associated hyperinflammatory response analogous to that observed in TB patients [19]. Type 1 and 2 interferon stimulation increases angiotensin-converting enzyme 2 (ACE2), the receptor for severe acute respiratory syndrome coronavirus 2 (SARS-CoV-2), expression on respiratory epithelial cells. In particular, lungs of patients with HIV-associated TB show the highest expression of ACE2 in epithelial cells compared with lungs of patients with TB only and healthy lungs [20]. Prior to antiretroviral therapy, HIV-1 infection also induces an interferon signaling gene (ISG) profile that is similar to that of TB patients, a pattern also implicated in the context of HIV-TB coinfection, even prior to TB symptom onset [16]. Given these findings, it is plausible that individuals living with HIV-1 and/or TB infection could be at increased risk of SARS-CoV-2 infection not only via immunosuppression but also via increased ACE2 expression, predisposing to enhanced viral uptake. This theory is supported by a small observational study of 46 COVID-19 patients in China. In that study, it was found that active and latent TB infection was predisposed to more severe presentations of COVID-19 as well as faster progression to clinical severity and that TB infection was more common in COVID-19 pneumonia cases than in other cases of bacterial and viral pneumonia within the same hospital [21]. Another recent report documents the coincidence of COVID-19 and TB [22].

Another aspect suggested by the pneumonia elicited by SARS-CoV-2 infection is the capacity to enhance TB progression in subclinical and latently infected individuals. The SARS-CoV-2 immunological milieu, dominated by interleukin (IL)-1β, IL-6, and tumor necrosis factor, is determined by single-cell RNA sequencing to be orchestrated by inflammatory monocytes that differentiate into various macrophage sublineages, with the proportional representation of sublineages defining the difference between patients with mild or severe disease development [19]. Bronchoalveolar lavage from patients who developed severe pneumonia possessed a unique macrophage population that expressed high levels of inflammatory chemokines (CCL2, CCL3, CCL4, CXCL9, CXCL10, CXCL11) and ISGs (APOBEC3A, ISG15, ISG20, GBP1, GBP5, IFITM3, MX1), which are similarly upregulated in TB and HIV-TB patients [16]. Thus, the lung immunological milieu elicited in COVID-19 patients has the potential to exacerbate TB immunopathogenesis in those with latent TB infection and increase their risk of TB progression.

Last, neutrophil activation in the form of release of neutrophil extracellular traps (NETs) has also been suggested to contribute to the severe pneumonia of COVID-19. Neutrophilia also predicts poor outcome in patients with COVID-19. In autopsy samples from 3 COVID-19 patients, neutrophil infiltration into pulmonary capillaries with fibrin deposition and extravasation into the alveolar space was observed [23]. In addition, the sera of patients with COVID-19 has elevated levels of myeloperoxidase-DNA and citrullinated histone H3; both are specific markers of NET formation [24]. Thus, the factors that underly neutrophilic skewing of the transcriptomic response of diabetics to TB may also contribute to severe pathology in COVID-19.

What are the practical consequences? Lower- and middle-income countries have yet to experience the full brunt of the COVID-19 epidemic. Yet, many already have the rising coincidence of DM and endemic TB. Routine services for both diseases have been disrupted by the initial necessarily vertical approach to COVID-19 control. Good glycemic control should be emphasized in known diabetics as well as stringent social distancing and shielding as necessary. There is no global policy on the treatment of latent TB in diabetics: a priority question now urgently in need of evidence. Longitudinal clinico-epidemiologic studies or registers of overlap among DM, COVID-19, and TB are pressing to advise risk and the potential need to change policy.

## Notes

***Disclaimer.*** The views in this article are the author’s alone and do not reflect the views of the agencies listed in the Financial Support section.

***Financial support.*** R. J. W. is supported by the Francis Crick Institute, which receives funding from Wellcome (FC0010218), UK research and innovation (FC0010218), and Cancer Research UK (FC0010218). He also receives support from Wellcome (014803 and 203135), National Institutes of Health (R01AI145436), and European and Developing Countries Clinical Trials Partnership (RIA2017T-2004).

***Potential conflicts of interest.*** The author: No reported conflicts of interest. The author has submitted the ICMJE Form for Disclosure of Potential Conflicts of Interest.
